# Development of pH-Sensitive Magnetoliposomes Containing Shape Anisotropic Nanoparticles for Potential Application in Combined Cancer Therapy

**DOI:** 10.3390/nano13061051

**Published:** 2023-03-15

**Authors:** Ana Rita F. Pacheco, Beatriz D. Cardoso, Ana Pires, André M. Pereira, João P. Araújo, Violeta M. Carvalho, Raquel O. Rodrigues, Paulo J. G. Coutinho, Teresa Castelo-Grande, Paulo A. Augusto, Domingos Barbosa, Rui A. Lima, Senhorinha F. C. F. Teixeira, Ana Rita O. Rodrigues, Elisabete M. S. Castanheira

**Affiliations:** 1 Physics Centre of Minho and Porto Universities (CF-UM-UP), University of Minho, Campus de Gualtar, 4710-057 Braga, Portugal; 2 Associate Laboratory LaPMET, 4710-057 Braga, Portugal; 3 Associate Laboratory LaPMET, 4169-007 Porto, Portugal; 4 IFIMUP—Instituto de Física dos Materiais, University of Porto, R. Campo Alegre, 4169-007 Porto, Portugal; 5 MEtRICs, Mechanical Engineering Department, University of Minho, Campus de Azurém, 4800-058 Guimarães, Portugal; 6 ALGORITMI Center, University of Minho, Campus de Azurém, 4800-058 Guimarães, Portugal; 7 Center for MicroElectromechanical Systems (CMEMS-UMinho), University of Minho, Campus de Azurém, 4800-058 Guimarães, Portugal; 8 LEPABE—Laboratory for Process Engineering, Environment, Biotechnology and Energy, Faculty of Engineering, University of Porto, Rua Dr. Roberto Frias, 4200-465 Porto, Portugal; 9 CEFT—Transport Phenomena Research Center, Faculty of Engineering, University of Porto, Rua Dr. Roberto Frias, 4200-465 Porto, Portugal

**Keywords:** combined cancer therapy, drug release, pH-sensitive magnetoliposomes, shape anisotropic nanoparticles, superparamagnetism

## Abstract

Late diagnosis and systemic toxicity associated with conventional treatments make oncological therapy significantly difficult. In this context, nanomedicine emerges as a new approach in the prevention, diagnosis and treatment of cancer. In this work, pH-sensitive solid magnetoliposomes (SMLs) were developed for controlled release of the chemotherapeutic drug doxorubicin (DOX). Shape anisotropic magnetic nanoparticles of magnesium ferrite with partial substitution by calcium (Mg_0.75_Ca_0.25_Fe_2_O_4_) were synthesized, with and without calcination, and their structural, morphological and magnetic properties were investigated. Their superparamagnetic properties were evaluated and heating capabilities proven, either by exposure to an alternating magnetic field (AMF) (magnetic hyperthermia) or by irradiation with near-infrared (NIR) light (photothermia). The Mg_0.75_Ca_0.25_Fe_2_O_4_ calcined nanoparticles were selected to integrate the SMLs, surrounded by a lipid bilayer of DOPE:Ch:CHEMS (45:45:10). DOX was encapsulated in the nanosystems with an efficiency above 98%. DOX release assays showed a much more efficient release of the drug at pH = 5 compared to the release kinetics at physiological pH. By subjecting tumor cells to DOX-loaded SMLs, cell viability was significantly reduced, confirming that they can release the encapsulated drug. These results point to the development of efficient pH-sensitive nanocarriers, suitable for a synergistic action in cancer therapy with magnetic targeting, stimulus-controlled drug delivery and dual hyperthermia (magnetic and plasmonic) therapy.

## 1. Introduction

The rapid and uncontrolled proliferation of tumor cells to adjacent tissues and organs, as well as the lack of targeted therapies with reduced systemic toxicity, make cancer one of the diseases with the highest mortality rate [1]. Several limitations associated with conventional treatments, specifically in the pharmacokinetic profile of the drug (e.g., reduced specificity, systemic toxicity and early metabolization and elimination) [2], point to an urgent need to develop new therapeutic approaches that allow the safe use of anticancer drugs.

In recent years, many studies have been focused on the development of magnetoliposomes to overcome the limitations of conventional liposomes [3,4,5]. These drug delivery systems, which consist of liposomes based on magnetic nanoparticles (MNPs), improve the therapeutic efficacy by the synergy between magnetic targeting and simultaneous hyperthermia and controlled drug delivery. In the presence of an external magnetic field gradient, the nanoparticles are responsible for guiding the nanosystem to the tumor site, where the content will be released on demand by using an alternating magnetic field (AMF) and/or a NIR laser light source. Moreover, this type of nanoparticle is able to produce a dual hyperthermia effect (magnetic and/or photothermal) [6,7,8,9].

Nanoparticles with superparamagnetic behavior, i.e., without coercivity, hysteresis or remanence, are ideal for biomedical applications. By adjusting their elemental composition and shape anisotropy, the magnetic properties of the MNPs can be optimized. Magnetite (Fe_3_O_4_) and maghemite (γ-Fe_2_O_3_) have been the most explored compositions for cancer therapy applications [10,11,12,13,14]. Recently, a high interest in the study of ferrites composed of alkaline earth metals has emerged. In particular, calcium and magnesium ferrites show high cell viability, as these elements are easily metabolized. Therefore, greater biocompatibility and biodegradability are guaranteed, as reported by several research groups [15,16,17,18,19]. Magnesium ferrite MNPs have been shown to have improved magnetic properties, acting as efficient hyperthermia agents [20]. Hirazawa et al. [21] reported that the partial replacement of Mg^2+^ ions by Ca^2+^ in magnesium ferrite nanoparticles (Mg_1-x_Ca_x_Fe_2_O_4_) favors biocompatibility, magnetization and thermal energy dissipation under an external magnetic field. The heating efficiency of nanoparticles with shape anisotropy, i.e., non-spherical, has shown clear advantages [22,23,24,25]. For instance, the easier magnetization along their longest axis gives them a longer blood circulation time and better magnetic and hyperthermia properties [26]. Unlike isotropic MNPs, their magnetic behavior results from the interaction between shape anisotropy and magnetocrystalline anisotropy [27,28].

Liposomes are advantageous nanosystems for drug delivery applications. As the non-covalent forces associated with their self-organization are reversible, this allows a dynamic transition between several morphologies in response to different stimuli [29]. At the tumor level, there are specific stimuli, which can be strategically used as triggers for controlled drug delivery [30]. For instance, variations in the pH value can stimulate the release of encapsulated therapeutic agents into the cytoplasmic space of abnormal cells through the endocytic pathway, by lysosomes and endosomes, using pH-sensitive liposomes [31]. As the bloodstream and healthy tissues have a neutral pH (approximately pH = 7.4), upon reaching the tumor microenvironment, which is more acidic (pH around 5), these types of formulations undergo a membrane destabilization, which helps the fusion of liposomes and drug release at acidic pH. At the endosomal level, these liposomes lose their initial stability due to their increased fusogenic potential, which prevents lysosomal degradation by enzymatic action, releasing the therapeutic compound into the cytosol [30].

Typically, pH-sensitive liposomes are composed of phospholipids of phosphatidylethanolamine (PE) and derivatives containing carboxylic groups that promote the stabilization of liposomes at neutral pH [32]. These types of natural and unsaturated phospholipids have fusogenic properties, as they have a good ability to adhere to cell membranes. This adhesion is ensured by the weak hydration of the small polar heads [32]. Several studies refer to the use of dioleoylphosphatidylethanolamine (DOPE) for the formation of pH-sensitive lipid vesicles. This phospholipid has an inverted conical shape and packing parameter above 1, naturally forming inverted structures [33,34,35]. The phospholipid dipalmitoylphosphatidylethanolamine (DPPE) has a conical geometry similar to DOPE; however, it is saturated, presenting a more rigid structure [36]. Liposomes containing only DOPE or DPPE have a reduced stability. To overcome this problem, it is usual to add an amphiphilic molecule, which stabilizes the lipid bilayer, e.g., cholesterol and derivatives such as cholesteryl hemisuccinate (CHEMS) [37]. In liposomes containing phospholipids with the PE group and CHEMS, the factor that dictates the behavior and conformation of the vesicle is the ionization state of CHEMS. At neutral pH, CHEMS is ionized (negatively charged), so the liposome presents a linear conformation as a result of electrostatic repulsions between the phosphate groups of DOPE/DPPE and the carboxylic group of CHEMS. At acidic pH, the CHEMS carboxylic group is protonated, which causes a change in the conformation of DOPE/DPPE, resulting in an inverted hexagonal phase and, consequently, the destabilization of vesicle membranes and the release of antineoplastic agents [30]. As it acts to reduce the transition temperature between the lamellar and hexagonal phases and decreases the permeability of biomembranes, preventing the early release of encapsulated drugs, cholesterol (Ch) was also included in the final lipid composition [30,37].

The present work focuses on the development of pH-sensitive nanosystems with improved magnetic properties derived from anisotropic-shaped MNPs. The structural, magnetic and hyperthermia characterization confirmed the synthesis of anisotropic mixed ferrite nanoparticles (Mg_0.75_Ca_0.25_Fe_2_O_4_) with high magnetization and thermal energy dissipation (under AMF or NIR irradiation). These were effectively surrounded by a pH-sensitive lipid bilayer of DOPE:Ch:CHEMS (45:45:10), forming solid magnetoliposomes. Overall, promising results were obtained for a controlled delivery of DOX under pH trigger, which was corroborated by cell viability assays performed on the HepG2 adherent human liver hepatocellular carcinoma cell line. To our knowledge, the combination of shape anisotropy magnesium/calcium ferrite nanoparticles and pH-sensitive liposomes of this nanosystem was not previously reported, showing promising results for application in combined therapies.

## 2. Materials and Methods

All the solutions and synthesis procedures were prepared using ultrapure water of Milli-Q grade (MilliporeSigma, St. Louis, MO, USA) and spectroscopic grade solvents.

### 2.1. Preparation of Anisotropic Nanoparticles of Mg_0.75_Ca_0.25_Fe_2_O_4_

Shape anisotropic magnetic nanoparticles of magnesium ferrite with 25% replacement by calcium ions (Mg_0.75_Ca_0.25_Fe_2_O_4_) were prepared using an adapted protocol previously described by Cardoso et al. [23]. To synthesize MNPs with anisotropic shape, several surfactants can be used as shape inducing agents [38]. In this protocol, a binary surfactant system was applied using octadecene and oleic acid as the solvent and shaping agent, respectively. The last one acts as a stabilizer and promotes the formation of specific arrangements on the surface in the nucleation and crystal growth phases of non-spherical nanoparticles [39].

For this purpose, a solution of 2 mM of iron (III) citrate tribasic monohydrate in 15 mL of octadecene was heated to 120 °C, under continuous magnetic stirring, until dissolution of the metallic precursor. Then, 0.25 mM of calcium acetate hydrate, 0.75 mM of magnesium acetate tetrahydrate and 3.1 mM of oleic acid were added to the previous solution. After 1 h, under magnetic stirring at 120 °C, a condenser was linked to the system to ensure a flow capable of keeping the molecules in the liquid state at controlled temperature. The mixture was heated at a rate of 1 °C per min, until reaching 200 °C. Thenceforth, a new increase in temperature was imposed at 5 °C per min until reaching the boiling point of octadecene (290 °C), remaining at this temperature for 1 h. The resulting MNPs were suspended in tetrahydrofuran (THF) and washed with water and ethanol in several cycles of magnetic decantation. Finally, half of the nanoparticles were calcined at 350 °C under an ultrapure nitrogen flow for 30 min to remove surface organic residues of octadecene and oleic acid. Both calcined and non-calcined MNPs were characterized to compare their properties. The application of this protocol is expected to yield cubic-shaped magnetic nanoparticles.

### 2.2. Preparation of Liposomes

In this work, pH-sensitive liposomes were prepared. Different lipid formulations were studied using the lipids dioleoylphosphatidylethanolamine (DOPE, from Avanti Polar Lipids, Birmingham, AL, USA), dipalmitoylphosphatidylethanolamine (DPPE, from Sigma-Aldrich, St. Louis, MO, USA), cholesterol (Ch, from Sigma-Aldrich, St. Louis, MO, USA) and cholesteryl hemisuccinate (CHEMS, from Sigma-Aldrich, St. Louis, MO, USA) at the molar ratios DOPE:CHEMS (7:3), DOPE:Ch:CHEMS (45:45:10) and DPPE:Ch:CHEMS (45:45:10). Liposomes composed of these formulations were synthetized following the ethanolic injection method [40], where an ethanolic lipid solution (1 × 10^−3^ M) was initially prepared according to the desired proportion. The volume of the lipid solution equivalent to this concentration was evaporated under an ultrapure nitrogen flow and subsequently redissolved in absolute ethanol in the same volume. Finally, the ethanolic solution was injected, drop by drop and under vortexing, to 3 mL of ultrapure water, inducing the formation of liposomes. The injection was performed while ensuring that the aqueous medium was at a higher temperature than the main phospholipid phase transition temperature (−16 °C for DOPE and 63 °C for DPPE) [41].

### 2.3. Preparation of Solid Magnetoliposomes

Solid magnetoliposomes loaded with doxorubicin were prepared following the method described in Ref [23]. For this purpose, the lipids DOPE, Ch and CHEMS were used at the molar ratio of 45:45:10, respectively. According to Ref [23], a thin lipid film with 2/3 of 1 mM of DOPE:Ch:CHEMS was prepared by solvent evaporation under a nitrogen flow. To this film, 3 mL of heptane was added, and this solution was ultrasonicated at 190 W for 10 min. In this step, reverse micelles with uniform sizes are formed. Afterward, 1 mM of dried MNPs was added, and the solution was again ultrasonicated for another 5 min to force its entry into the previously formed micelles. To promote the nanosystem precipitation, this solution was placed in the cold. Then, by magnetic decantation, reverse micelles containing the MNPs were purified, and the resultant pellet was completely dried through an ultrapure nitrogen flow. Finally, the pellet was resuspended in 3 mL of ultrapure water, and a solution containing the remaining lipid and DOX (1.13 × 10^−4^ M) was added by ethanolic injection, under vortex, forming the second lipid layer and consequently, the solid magnetoliposomes.

### 2.4. Spectroscopic Measurements

Spectroscopic measurements were carried out at Photophysics Laboratory of the Physics Center of the University of Minho, Braga, Portugal. Absorption spectra of MNPs dispersions were acquired on a double-beam Shimadzu UV-Vis-NIR spectrophotometer, model UV-3600 Plus (Shimadzu Corporation, Kyoto, Japan). The fluorescence emission spectra of samples containing the SMLs were measured on a Fluorolog 3 spectrofluorometer (HORIBA Jobin Yvon IBH Ltd., Glasgow, UK) equipped with double monochromators in excitation and emission, exciting at 480 nm (DOX excitation).

### 2.5. Structural Characterization

The crystalline structure and phase identification of the shape anisotropic magnetic nanoparticles were determined by X-ray diffraction (XRD), using a PAN’alytical X’Pert PRO diffractometer (Malvern Panalytical Ltd., Malvern, UK) in a Bragg–Brentano configuration operating with Cu K_α_ radiation (λ = 0.154060 nm), at the Electron Microscopy Unit of the University of Trás-os-Montes and Alto Douro (UTAD), Vila Real, Portugal.

All images of Mg_0.75_Ca_0.25_Fe_2_O_4_ MNPs and SMLs were obtained by transmission electron microscopy (TEM), using a JEOL JEM-1010 high-contrast microscope operating at 100 kV (Centro de Apoio Científico-Tecnolóxico à Investigación (CACTI), Vigo, Spain). The samples were ultrasonicated and deposited on copper grids with carbon and Formvar. The TEM images processing was performed using ImageJ software (version 1.53t, National Institutes of Health (NIH), Bethesda, MD, USA). The manual selection of the nanoparticles’ diameter allowed estimating their average size. The histograms obtained by this analysis were fitted to a Gaussian distribution.

The average hydrodynamic diameter and zeta potential (ζ) of the developed liposomal formulations and SMLs were measured on a dynamic light scattering (DLS) equipment Litesizer^TM^ 500 from Anton-Paar (Anton-Paar GmbH, Graz, Austria) equipped with a laser diode of λ = 658 nm. For liposomal measurements, aqueous solutions of 0.3 mM were prepared in phosphate buffer pH = 7.4 and pH = 5. In the case of SMLs, aqueous solutions of 0.5 mM were measured in aqueous buffer solutions. Before measurement, all samples were sonicated and filtered using a Socorex Dosys^TM^ all-glass syringe attached to a hydrophilic polytetrafluoroethylene (PTFE) filter. The analysis was carried out at 25 °C using an Univette cell with an optical path of 10 mm. Three independent measurements were performed for each sample. For SMLs colloidal stability assays, aqueous solutions in PBS pH = 7.4 were prepared and stored at 4 °C for 7 days. Differences in the hydrodynamic diameter, PDI and zeta potential were recorded by DLS and ELS, as described above.

### 2.6. Magnetic Characterization

The magnetic properties of the mixed ferrite nanoparticles were evaluated on a MPMS3 Superconducting Quantum Interference Device (SQUID) magnetometer Quantum Design MPMS5XL (Quantum Design Inc., San Diego, CA, USA) at IFIMUP (University of Porto, Porto, Portugal). The hysteresis loop was obtained by measuring the magnetization of the samples as a function of the applied magnetic field (H), applying fields up to 5.5 T, at room temperature (300 K). For that, the temperature was fixed, and the magnetic moment (M) was measured at different values of H.

### 2.7. Hyperthermia Measurements

The heat capability generation of the nanoparticles was quantified through the specific absorption rate (SAR), described by Equation (1):

(1) $$\mathrm{SAR}=C\;\frac{\Delta T}{\Delta t}\times \frac{m_{s}}{m_{m}}\;,$$

where C is the specific heat capacity of the medium (4.186 J g^−1^ K^−1^); ΔT/Δt is the initial slope of the temperature curve as a function of time; and $m_{s}$ and $m_{m}$ are the mass of the solvent and of the magnetic material, respectively [42]. As SAR depends on the frequency and intensity of the applied AMF, it does not allow comparing values with confidence. Thus, the intrinsic loss power (ILP, nH·m^2^/kg) is the parameter designed for this purpose, according to Equation (2):

(2) $$\mathrm{ILP}=\frac{\mathrm{SAR}}{H^{2}f}$$

where H corresponds to the intensity of the magnetic field (kA/m), and f is the frequency (kHz) [43].

#### 2.7.1. Magnetic Hyperthermia

The heating capabilities of Mg_0.75_Ca_0.25_Fe_2_O_4_ nanoparticles, in the presence of an alternating magnetic field, were evaluated using a hyperthermia setup at the Faculty of Engineering of University of Porto (Porto, Portugal) working at f = 155 kHz and H = 8.5 kA/m. Temperature variations resulting from the application of an AMF were recorded for 30 min. In the absence of magnetic field, the cooling of the solutions was also recorded for half an hour. For the magnetic hyperthermia measurement, the SAR value was calculated following Equation (1) and using the initial linear slope method. This indicates the rate at which electromagnetic energy is absorbed per unit mass of MNPs.

#### 2.7.2. Photothermal Hyperthermia

The potential of anisotropic MNPs in photothermal therapy (PTT), i.e., their ability to dissipate thermal energy under NIR radiation, was evaluated using an experimental irradiation setup at the Physics Centre of University of Minho. The developed setup consists of a sample holder, a laser light source with a wavelength of 808 nm and 1 W/cm^2^ of power density, and a T-type thermocouple connected to a digital multimeter (Agilent U1242A) for temperature measurement. Before each measurement, the temperature was stabilized at room temperature, and then, the samples were irradiated. Temperature variations were recorded over time for 30 min, and afterward, the laser was turned off, and cooling was recorded in the same way. The SAR calculation was performed as described in the previous section.

### 2.8. Drug Encapsulation Efficiency

Doxorubicin encapsulation efficiency, EE(%), in magnetoliposomes was estimated by fluorescence spectroscopy. Initially, the fluorescence emission spectra of aqueous solutions of DOX with different concentrations were measured. Using the maximum fluorescence intensity of each spectrum, obtained with excitation at 480 nm, a calibration curve of fluorescence intensity was plotted as a function of DOX concentration, and then, a linear regression was fitted. To determine the value of EE(%), 1.5 mL of SMLs solution was placed on the top of Amicon^®^ Ultra centrifugal filter units (100 kDa), which were subjected to centrifugations, at 3000 rpm for 10 min. As a result, two phases were obtained: the solution retained above the membrane of the filters, corresponding to doxorubicin, which was effectively encapsulated in the SMLs, and the aqueous solution below the membrane, containing the non-encapsulated drug. Subsequently, and based on the calibration curve initially obtained, the concentration of non-encapsulated drug was quantified. Three independent measurements were performed, and the respective standard deviation (SD) was calculated. The last step consisted of calculating the EE(%) of DOX in SMLs, as described by Equation (3):

(3) $$\mathrm{EE}\left(\%\right)=\frac{{\left[\mathrm{Drug}\right]}_{\;\mathrm{total}}-{\left[\mathrm{Drug}\right]}_{\;\mathrm{non}-\mathrm{encapsulated}}}{{\left[\mathrm{Drug}\right]}_{\;\mathrm{total}}}\times 100$$

### 2.9. Drug Release Kinetics

The DOX release profile of the developed nanosystems was evaluated using two different media, one to simulate the acidic tumor microenvironment (pH = 5) and another for physiological conditions (pH = 7.4). For this, solid magnetoliposomes were diluted in phosphate buffer with the desired pH value, and these solutions were placed above the Amicon filter membrane. The volume below the cellulose membrane was filled with 7.5 mL of the corresponding buffer solution, and the Amicon filters were placed on an orbital shaker at 300 rpm. Aliquots of 200 µL were pipetted out from the solution placed below the membrane, always being replaced with 200 µL of PBS at the corresponding pH. This procedure was repeated for 48 h. For each aliquot, the fluorescence spectrum was measured, with excitation at 480 nm, and the concentration of the released doxorubicin was quantified using the calibration curves obtained in PBS 5 and 7.4, as described in Section 2.8. Three independent measurements were performed. To better understand the kinetics, the cumulative DOX release curves were fitted to the Weibull and first-order models using Prism 8 software (GraphPad Software, La Jolla, CA, USA).

### 2.10. Cell Culture

The adherent human liver hepatocellular carcinoma HepG2 cell line (ATCC HB-8065—American type culture collection, Virginia, VA, USA) was cultured and maintained in T75 culture flasks with Roswell Park Memorial Institute (RPMI) 1640 medium, with GlutaMAX™ supplemented with 10% fetal bovine serum (FBS), 1% Penicillin-Streptomycin in a humidified incubator, at 37 °C, with 5% CO_2_ environment. When confluence was achieved, the sub-culturing process was performed by trypsinization, and the cell count was performed using Trypan blue and a Neubauer chamber.

### 2.11. Biological Studies

For viability assays, the AquaBluer assay (MultiTarget Pharmaceuticals, LLC) was used. HepG2 cells were seeded on 96-well plates, at a density of 7000 cells/well, and were incubated with supplemented RPMI-1640 medium overnight, at 37 °C, in the CO_2_ incubator. Afterward, the medium was aspirated, and the viability assay was conducted by adding 100 μL of AquaBluer solution (1:100 in supplemented RPMI-1640 medium) to each well and incubation for 2 h, at 37 °C, under 5% CO_2_ environment. Then, a microplate reader at λ_exc_ = 540 nm and λ_em_ = 590 nm wavelengths was used to evaluate the viability. This process was repeated after 48 h of adding DOX-loaded SMLs at two different DOX concentrations (5.65 × 10^−5^ M and 1.13 × 10^−4^ M), as well as free DOX with the respective concentrations. Untreated HepG2 cells were used as a control. For each condition, three independent measurements were performed.

## 3. Results and Discussion

### 3.1. Characterization of Mg_0.75_Ca_0.25_Fe_2_O_4_ Nanoparticles

#### 3.1.1. UV-Vis-NIR Absorption Spectra

**Figure 1:**
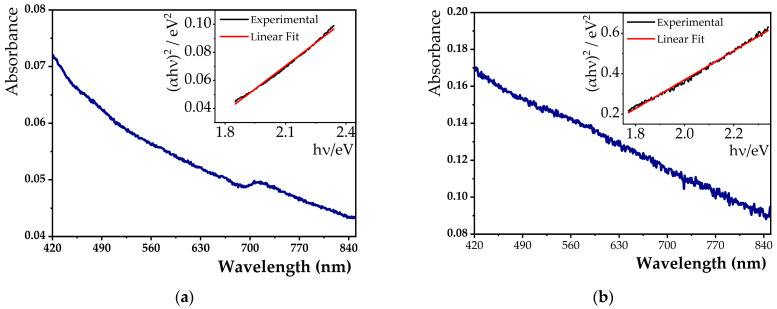
Absorption spectra of 0.2 mM aqueous solutions of non-calcined (**a**) and calcined (**b**) Mg_0.75_Ca_0.25_Fe_2_O_4_ nanoparticles, using ultrapure water as a reference. Inset: Tauc plot fitted to the experimental data.

The optical properties of Mg_0.75_Ca_0.25_Fe_2_O_4_ nanoparticles (with and without calcination), represented by their absorption spectra in Figure 1, show that both ferrites exhibit a broad absorption band in the visible region, which is consistent with the blackish color of the MNPs dispersion.

Through the obtained spectra, it was possible to determine the energy band gap using a Tauc plot, given by Equation (4):

(4) $${\left(\alpha h\nu \right)}^{n}\;\sim \;\left(h\nu -E_{g}\right)$$

where α designates the absorption coefficient, h corresponds to Planck’s constant, ν is the frequency, n is an exponent dependent on the nature of the transition (if n = 2, it corresponds to a direct semiconductor; when n = 1/2, it is an indirect semiconductor), and $E_{g}\;$corresponds to the energy band gap [44]. For both mixed calcium/magnesium ferrites, linear relations were obtained for n = 2, indicating that they behave as direct semiconductors. Through the intersection of (αhν)^2^ linear extrapolation with the Y-axis, the MNPs energy band gap was estimated, with a value of 1.46 eV and 1.48 eV for non-calcined and calcined nanoparticles, respectively. The obtained band gaps are slightly higher than those previously obtained for cubic nanoparticles with the same composition (1.29 eV) [23]. In another study, reported by the same research team, it was reported that the percentage of calcium is directly proportional to the band gap. For Mg_0.75_Ca_0.25_Fe_2_O_4_ MNPs, a value of $E_{g}$ = 1.51 eV was estimated [45], similar to the ones determined in the present work.

#### 3.1.2. Crystalline Structure

**Figure 2:**
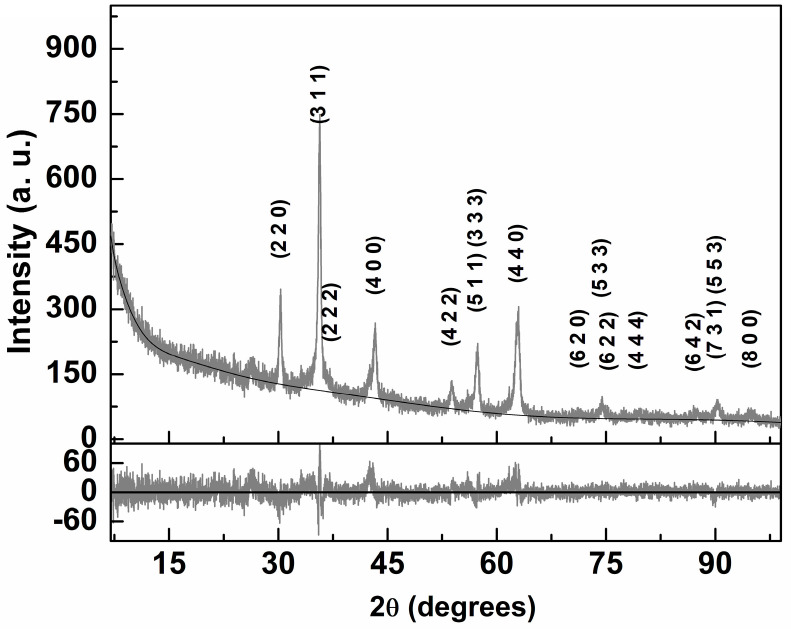
X-ray diffractogram of calcined Mg_0.75_Ca_0.25_Fe_2_O_4_ nanoparticles and corresponding Rietveld analysis with Miller indices. Bottom panel: Residuals of the fitting procedure.

XRD diffraction pattern is essential in the characterization of MNPs, as it provides information about their crystalline structure, average grain size and degree of purity. As the thermal treatment promotes an enhanced crystallinity of nanoparticles, the removal of surface organic impurities and favors the crystals’ growth, facilitating the achievement of a crystalline phase [46], the calcined MNPs were selected for analysis by X-ray diffraction, according to the Rietveld method. The respective XRD diffractogram is shown in Figure 2 and was analyzed using Profex software (version 4.3.6) [47], which uses Rietveld-type calculations implemented by BGMN [48].

From the crystallographic open database (COD), the CIF file with number 1011245 (space group Fd-3m:1) was imported and processed by Profex. However, it was necessary to make changes in the unit cell composition, so that 25% of the positions occupied by magnesium ions were replaced by calcium ions, and the distribution of cations across the tetrahedral and octahedral sites was considered to be in accordance to the mixed ferrite stoichiometry. Further, the ferrite unit cell also included the inversion degree. All the experimental diffraction peaks could be indexed to the ferrite spinel phase, pointing to a high purity of the obtained magnetic nanoparticles. These peaks occur at 18.2° (1 1 1), 30.0° (2 2 0), 35.4° (3 1 1), 37.0° (2 2 2), 43.1° (4 0 0), 47.2° (3 3 1), 53.5° (4 2 2), 57.0° (5 1 1), 57.0° (3 3 3), 62.6° (4 4 0), 65.9° (5 3 1), 71.1° (6 2 0), 74.1° (5 3 3), 75.1° (6 2 2), 79.1° (4 4 4), 82.1° (7 1 1), 82.1° (5 5 1), 87.0° (6 4 2), 89.9° (7 3 1), 89.9° (5 5 3), 94.7° (8 0 0) and 97.7° (7 3 3). A good fit was obtained, with χ^2^ = 1.46 and R_P_ = 8.67, when the inversion degree was fixed at a value of 0.825, which was previously reported for magnesioferrite [49]. The obtained lattice parameter was 8.367 Å, which is near to the value reported in the magnesioferrite CIF file (8.360 Å). In order to adequately describe the relative intensity and shape of the various diffraction peaks using BGMN [48], it was required to consider the size broadening effects, Debye–Waller factors (TDS) and preferred orientation (PO). The latter is taken into account in accordance with the ferrite space group through spherical harmonics. The main results of the XRD analysis are indicated in Table 1. If the inversion factor is fitted, only a small improvement is obtained, resulting in χ^2^ = 1.44 and R_P_ = 8.62 with an inversion degree of 0.675. The fact that the fit is not perfect, especially in the main (3 1 1) and in (4 0 0) diffraction peaks, points to the influence of the anisotropic shape of the prepared nanoparticles. An average size of 18.7 ± 0.3 nm is estimated using all the diffraction peaks through their size broadening effects, as implemented in BGMN [48,50]. This value is similar to others reported in the literature for particles with the same composition [51]. The instrument peak broadening was taken into account through Profex software, using the specific characteristics of the XRD equipment. Interestingly, the need to consider strain effects on the fit of the diffractogram points to a larger shape anisotropy than that of the magnetic nanoparticles previously obtained with the same expected composition [23].

#### 3.1.3. Size and Shape Analysis

Considering the human physiology and its interaction mode with foreign systems, it is essential to study the influence of physicochemical properties of the nanosystem on normal organism functioning to avoid its opsonization and ensure cellular uptake [52]. For this purpose and for a rigorous determination of magnetic nanoparticles’ size distribution and morphology, characterization by TEM was performed. For in vivo applications, the nanosystems must have reduced sizes, up to 150 nm [53].

**Figure 3:**
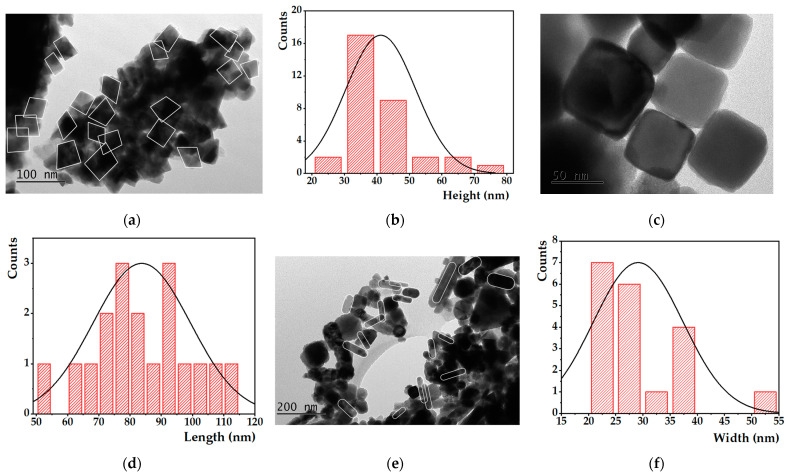
TEM images and size histograms fitted to a Gaussian distribution of non-calcined (**a**–**c**) and calcined (**d**–**f**) Mg_0.75_Ca_0.25_Fe_2_O_4_ nanoparticles, at different magnifications.

Through the TEM images presented in Figure 3, it is verified that the non-calcined nanoparticles have a cubic shape. However, it is highlighted that thermal treatment can lead to a shape transformation of the synthesized anisotropic MNPs, developing a more elongated nanorod-like structure, with recrystallization and grain growth over time. Possibly, given their composition and crystallinity, this type of MNPs are more susceptible to different pressure and temperature conditions, presenting this morphological change apparently favorable in nanomedicine, since higher surface area/volume ratio is reflected in larger nanoparticles’ magnetization [26].

On the one hand, and using the size histogram and Gaussian distribution (Figure 3b) fitted to the experimental data in Figure 3a,c, average sizes of 41 ± 11 nm were obtained, with some larger nanocubes being observed in Figure 3c. On the other hand, considering the elongated MNPs identified in Figure 3e, an average length and width of, respectively, 89 ± 26 nm (Figure 3d) and 29 ± 8 nm (Figure 3f) were determined. The nanorods’ size distribution is much more heterogeneous compared to the size distribution for cubic-shaped ferrites. This occurs because the calcined MNPs are in different stages of elongation, which is reflected in the Gaussian distributions presented in Figure 3d,f.

#### 3.1.4. Magnetic Properties

For biomedical applications, it is essential to ensure that the magnetic core of SMLs has ideal magnetic characteristics for both magnetic guidance and hyperthermia. To confirm this, the dependence of the nanoparticles’ magnetization (M) on the applied field (H) was evaluated by SQUID.

**Figure 4:**
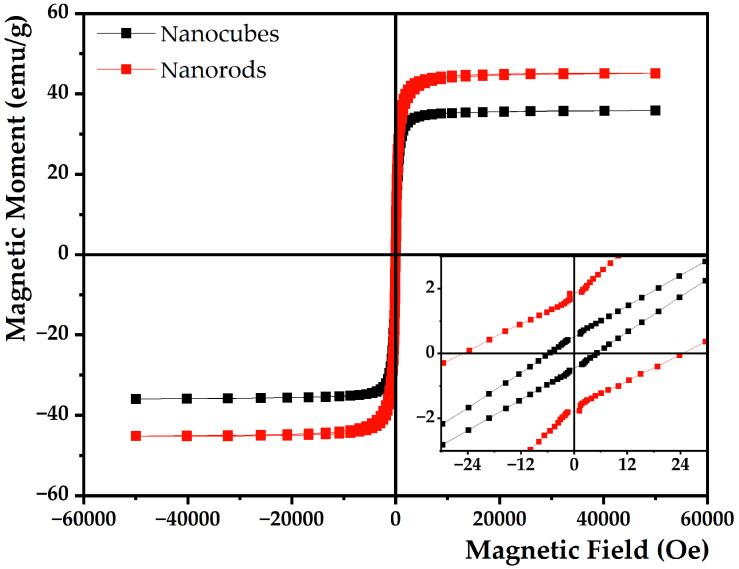
Hysteresis cycle of non-calcined (black) and calcined (red) Mg_0.75_Ca_0.25_Fe_2_O_4_ nanoparticles. Inset: Enlargement of M–H curve in the low field region.

The hysteresis cycles of Mg_0.75_Ca_0.25_Fe_2_O_4_ magnetic nanoparticles, at room temperature, presented in Figure 4 allowed a determination of the values of coercivity (H_c_), remnant magnetization (M_r_), saturation magnetization (M_s_) and calculation of the ratio M_r_/M_s_ (squareness value), summarized in Table 2. The almost closed hysteresis loop and the low values of remnant magnetization exhibited by both types of nanoparticles (0.52 emu/g for nanocubes and 1.74 emu/g for calcined nanoparticles) are compatible with superparamagnetic behavior. Furthermore, the squareness value below 0.1 in both MNPs shows that more than 90% of the magnetization is lost after removing the external magnetic field [54]. This proves that these particles exhibit superparamagnetic behavior at 300 K. Moreover, coercivity must be low and close to zero, which is verified in both ferrites. Without residual magnetization after the removal of magnetic field, the agglomeration of nanoparticles is avoided, consequently increasing their circulation time in the body [55]. Both ferrites showed an improved magnetism compared to the spherical ones with an equal proportion of magnesium and calcium ions (12.98 emu/g) [45]. Hence, it is concluded that shape anisotropy favors the magnetism of the MNPs and therapeutic effectiveness. In addition, calcined nanoparticles exhibit higher magnetization, therefore being the most promising (M_s_ = 45.21 emu/g), pointing to the development of a new and efficient strategy to synthetize rod-shaped mixed ferrites with better magnetic properties. Given their magnetic behavior, they are promising for magnetic guidance of the nanosystem to the target region and as hyperthermia agents.

#### 3.1.5. Hyperthermia Capacity

To evaluate their potential in magnetic and photothermal hyperthermia, the heating capabilities of nanoparticles with anisotropic shape were assessed under the application of an alternating magnetic field or under a laser light source, respectively. As mentioned in Section 3.1.2., only the calcined batch of MNPs was evaluated, since these MNPs have better magnetization derived from the thermal treatment at 350 °C.

**Figure 5:**
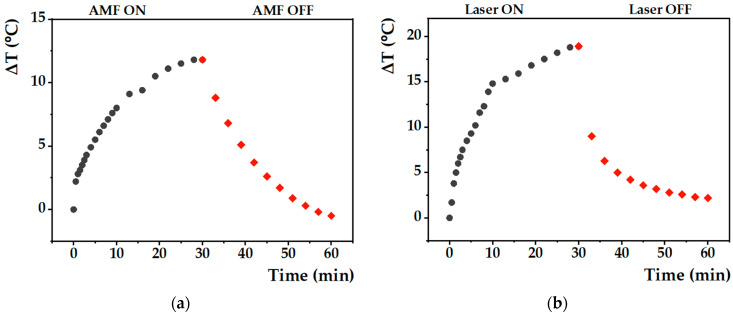
Heating profile (gray: heating; red: cooling) of the aqueous solutions of calcined Mg_0.75_Ca_0.25_Fe_2_O_4_ nanoparticles under exposure to: (**a**) AMF with 11 mT amplitude and frequency of 155 kHz; (**b**) laser light source with 808 nm wavelength and 1 W/cm^2^ power density.

Several published studies demonstrate that magnetic nanoparticles with anisotropic shape (e.g., cubic and elongated) have higher SAR values compared to spherical MNPs [56,57]. The heating capability of cubic-shaped Mg_0.75_Ca_0.25_Fe_2_O_4_ nanoparticles under an alternating magnetic field was already evaluated by Cardoso et al. [23]. These nanoparticles exhibited an excellent temperature variation of 19.4 °C in 30 min with H = 200 Gauss and f = 688 kHz. In this work, to assess the potential of the synthesized MNPs, an aqueous suspension of the magnesium/calcium ferrite was evaluated under an AMF amplitude of 11 mT and frequency of 155 kHz (the product of the applied magnetic field and oscillating frequency is below the limit acceptable for use in small regions of the human body). The heating profile (Figure 5a) shows that there is a gradual and time-dependent increase in temperature, with a reasonable temperature variation of approximately 12 °C in 30 min. In cancer cells, this local temperature increase will induce a series of metabolic reactions, including programed cell death, essential to fight the disease. In order to determine the specific absorption rate, the initial linear slope method of the ΔT/Δt curve was used. The values of SAR and ILP calculated according to Equations (1) and (2), 203.27 W/g and 0.47 ± 0.12 nH·m^2^/kg, respectively, confirmed their potential as magnetic hyperthermia agents.

Although several studies already reported different nanomaterials as magnetic hyperthermia and photothermia agents [9,58,59], to the best of our knowledge, the capacity of shape anisotropic mixed ferrite nanoparticles in phototherapy was never reported. Furthermore, the potential of this type of ferrite in dual hyperthermia is also innovative in nanoparticle research for combination therapies. In Figure 1, it is observed that the synthesized nanoparticles absorb at λ = 808 nm, allowing a study of their potential in PTT. As described in the literature, it is usual to apply a minimum laser power density of 1 W/cm^2^ [7,8,9,24,58]. In this way, we reproduced the common analysis conditions using a NIR laser with the same power density. Thus, the magnetic nanoparticles were exposed to the NIR light source, and the results of temperature variation as a function of time are displayed in Figure 5b. When the laser wavelength is in resonance with the surface plasmonic frequency, part of that energy is dissipated/released as heat [60]. When irradiated for 30 min at λ = 808 nm, a high temperature variation of 18.9 °C was achieved. This heating effect can strongly reduce the viability of cancer cells and is sufficient to exert toxic effects on the tumor by PTT. The photothermal hyperthermia data obtained (with the initial slope method) are summarized in Table 3. A SAR value of 1.1 × 10^4^ W/g was obtained for anisotropic calcined MNPs, which compares well with the reported values for other ferrite nanoparticles [60] (Table S1 in Supplementary Material). The high SAR in both hyperthermia measurements, especially under a NIR laser, corroborates the potential of these MNPs as agents in cancer therapy.

### 3.2. Characterization of pH-Sensitive Magnetoliposomes

The calcined batch of Mg_0.75_Ca_0.25_Fe_2_O_4_ nanoparticles was selected for encapsulation in pH-sensitive magnetoliposomes, as these MNPs have the desired composition, an average diameter suitable for biological applications and good magnetization, which is essential to locate the nanosystem in the target region where it will act as hyperthermia agent. For that purpose, different liposomal formulations were studied by DLS and electrophoretic light scattering (ELS) for the selection of the best pH-sensitive behavior.

#### 3.2.1. Selection of the pH-Sensitive Liposomal Formulation

The best pH-sensitive formulation was selected after studying the variations of several lipid compositions with medium pH (pH = 5 to simulate the tumor microenvironment and pH = 7.4 to mimic physiological fluids). Analyzing in detail the results obtained by DLS and ELS (Table 4), i.e., the hydrodynamic size and zeta potential, it can be noted that the formulations composed of a phosphatidylethanolamine derivative as the main component are strongly sensitive to changes in medium pH. For liposome formulations containing DOPE or DPPE, the stability differences between acidic and basic media are significant. When combined with CHEMS—DOPE:CHEMS (7:3), DOPE:Ch:CHEMS (45:45:10), DPPE:Ch:CHEMS (45:45:10)—these changes are a result of the protonation of the carboxylic groups of the latter molecule at acidic pH values, which strongly compromises the stability of the lipid bilayer [30]. For this study, the polydispersity index (PDI) was also measured by DLS, which provides information about the degree of dispersion of the MNPs. Ideally, this index should take values lower than 0.3, reflecting uniform and monodisperse populations [61].

It is noted that at lower pH, the PDI increases, and the absolute value of the zeta potential decreases. This reveals that, when reaching a more acidic microenvironment, such as in tumors, the formulations can be destabilized, and their content release can be promoted. Liposomes of the DPPE:Ch:CHEMS (45:45:10) formulation had slightly higher sizes and PDI than what is desirable for body circulation (at pH 7.4), while the DOPE:Ch:CHEMS (45:45:10) formulation showed the greatest difference in zeta potential at both pH values. Thus, the DOPE:Ch:CHEMS (45:45:10) formulation stands out as the most promising for the development of pH-sensitive SMLs, being selected to encapsulate the calcined magnetic nanoparticles.

#### 3.2.2. Proof of SMLs Synthesis

**Figure 6:**
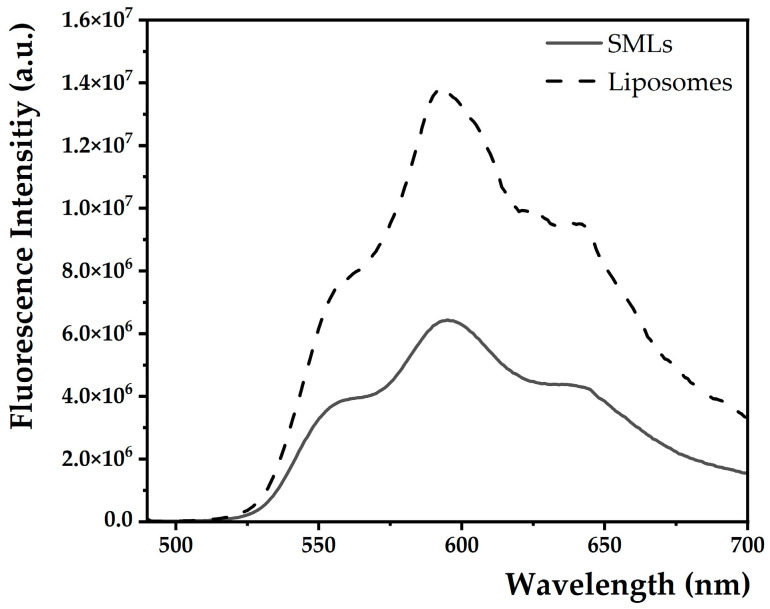
Fluorescence emission spectra (λ_exc_ = 480 nm) of doxorubicin (1.13 × 10^−4^ M) in DOPE:Ch:CHEMS (45:45:10) SMLs with a magnetic core (solid line) and in liposomes with the same formulation (dashed line).

Using fluorescence emission measurements, it was possible to prove that the shape anisotropic magnetic nanoparticles were efficiently surrounded by a lipid bilayer. Fluorescence emission spectra of doxorubicin in liposomes and SMLs loaded with the same concentration of DOX and with the same lipid concentrations confirm its encapsulation (Figure 6). In the SMLs aqueous solution, a reduction in DOX fluorescence intensity compared to the liposome sample is evident. This occurs because the MNPs present in the magnetic core of SMLs act as quenchers of the fluorescence emitted by DOX. This quenching effect results from the broad absorption spectrum of calcined mixed ferrites, which have the capacity to absorb part of the energy emitted by the fluorescent compound after its excitation at 480 nm. Still, this phenomenon can also be explained by photoinduced electron transfer, as well as an increase in intersystem crossing efficiency due to the heavy atom effect. The latter prevails in fluorescence inhibition in this type of nanosystem.

The magnetoliposomes prepared show suitable average sizes for combined therapy. Comparing the hydrodynamic diameter of DOPE:Ch:CHEMS liposomes (45:45:10) and of SMLs with the same composition at physiological pH (Table 5), it is verified that the liposomes have very slightly larger sizes. Furthermore, the lipid vesicles present a less negative zeta potential compared to solid magnetoliposomes, possibly due to the presence of MNPs near the bilayer surface, conferring an even more negative surface charge. In SMLs, the larger negative charge density on the surface (more negative zeta potential) may act to reduce the average diameter due to a decrease in aggregation [62]. Thus, the reductions in the hydrodynamic size, as well as in PDI and zeta potential, confirm the synthesis of solid magnetoliposomes and indicate suitable properties for biomedical applications, guaranteeing the enhanced permeability and retention (EPR) effect.

A TEM image of SMLs is presented in Supplementary Material (Figure S1), revealing roughly spherical structures, with a solid interior (the magnetic cluster) surrounded by a membrane. The image reveals some polydispersity in size, as inferred from DLS measurements (PDI near 0.2). It is clear that the SMLs are not aggregated.

Using DLS and ELS techniques, the colloidal stability of pH-sensitive SMLs in physiological medium was assessed (Figure S2 in Supplementary Material). The variation of the hydrodynamic diameter, PDI and zeta potential shows that the solid magnetoliposomes remain stable for at least 5 days. After 5 days, there was a slight increase in size from 176 nm to 235 nm, while the PDI and zeta potential presented favorable values during the assay. These results indicate that the prepared solid magnetoliposomes are generally stable for at least 5 days.

### 3.3. Photophysical Studies of Drug-Loaded Magnetoliposomes

#### 3.3.1. Doxorubicin Encapsulation Efficiency

The percentage of DOX encapsulated in the SMLs of DOPE:Ch:CHEMS (45:45:10) nanosystems containing Mg_0.75_Ca_0.25_Fe_2_O_4_ nanoparticles was estimated by fluorescence spectroscopy assays. Calculated using Equation (3), the obtained values of EE(%) are listed in Table 6. A high encapsulation efficiency was obtained (98.71% ± 0.97%), which points to a promising use of these new pH-sensitive SMLs in combined cancer therapy, using DOX as a chemotherapeutic drug.

#### 3.3.2. Doxorubicin Release Kinetics

**Figure 7:**
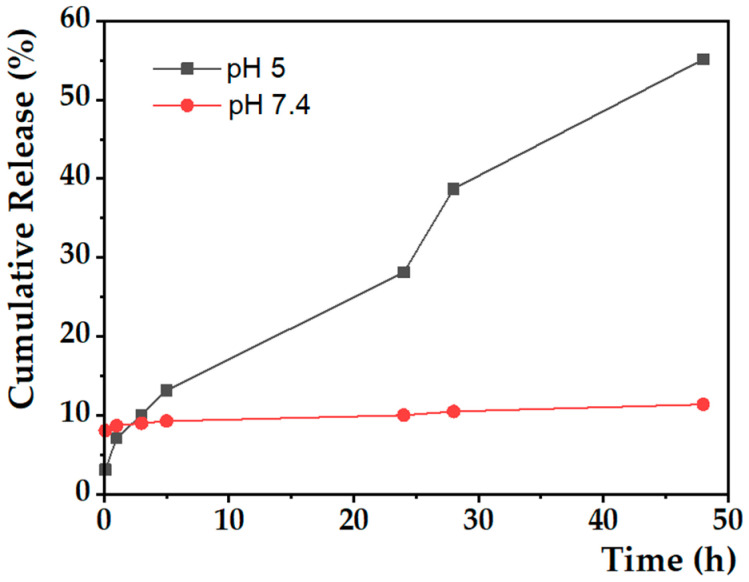
Cumulative release profiles (average of three replicas) of doxorubicin (%) over time (h) encapsulated in SMLs at pH = 5 (gray line) and pH = 7.4 (red line).

The release profile of doxorubicin from the SMLs was determined at pH = 5 and pH = 7.4 by fluorescence measurements. Figure 7 shows the strong dependence of the release rate on the medium pH. After 20 h, the drug release starts to be much more pronounced in acidic medium compared to that at physiological pH, reaching about 55% release after 48 h, while at pH = 7.4, a maximum of only 11% is achieved. On the one hand, at neutral pH, the DOX release percentage is almost invariable, showing that drug release is prevented. On the other hand, at pH = 5, an increased and continuous release is observed over time as a result of membrane destabilization at this pH. Furthermore, a saturation release was not achieved after 48 h, indicating that there is a gradual destabilization of the biomembrane, promoting the release of its content.

To better understand the release kinetics, two mathematical models were used to predict the release mechanism of the therapeutic agent. In this work, the cumulative DOX release curve was fitted to the Weibull model and first-order kinetics.

In the Weibull model, a distribution function determines the fraction of drug dissolved in solution (m) at time t (Equation (5)):

(5) $$m=1-\exp^{-{\left(t-T_{i}\right)}^{\frac{\alpha }{b}}}$$

where $T_{i}$ designates the release latency time (normally taking the value 0), and α describes the time scale of the process, enabling the determination of the shape of the release progression curve (b). When b = 1, the release kinetics is equivalent to an exponential function; if b > 1, the function is sigmoid constrained by an inflection point. In the case of b < 1, we are facing a parabolic-type function with a high initial slope [63]. An alternative model is the first-order kinetics, in which release profile is described by Equation (6):

(6) $$F\;\left(\%\right)={\;M}_{0}\times \left(1-e^{-\mathrm{kt}}\right)$$

where F is the percentage of released drug, $M_{0}$ is the total amount of drug, k is the first-order rate (variable), and t is the time [64].

**Figure 8:**
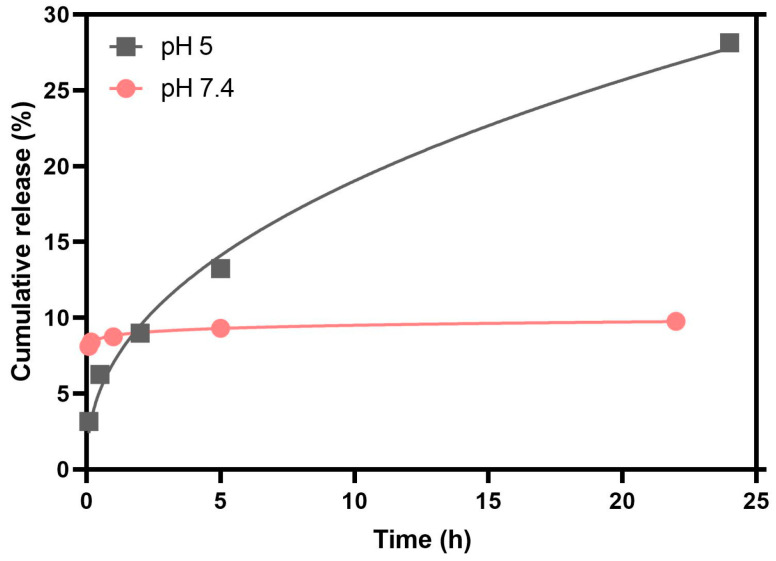
Cumulative release profile of doxorubicin (%) over time (h) encapsulated in SMLs at pH = 5 (gray line) and pH = 7.4 (red line), fitted to the Weibull model.

Both fittings were performed on the release kinetic data up to 24 h of measurement, showing good quality. The parameters obtained are listed in Table 7, and the determination coefficients demonstrate that the best model to describe the release profile is the Weibull model, for both pH values (Figure 8). Despite this, as it is an empirical model, the parameters α and b associated with the model have no physical meaning. In the literature, a correlation has already been established between parameter b and the release diffusional mechanism of the antitumor agent. When b ≤ 0.75, the diffusion is of the Fickian type, i.e., through Euclidian and fractal spaces; if 0.75 < b < 1, there is a combination of Fickian diffusion and Case II transport mechanisms. However, in situations where b takes values greater than 1, the release follows a complex mechanism [65]. Thus, the DOX diffusion mechanism at pH = 5 and pH = 7.4 is of the Fickian type. For the first-order kinetics, the parameter k reveals that, up to 24 h, the formulation exposed to a buffer solution at pH = 5 presents a release rate approximately 2.2 times higher than that obtained for neutral pH. These results validate the pH sensitivity of the magnetoliposomes developed, as well as their potential in application in oncological therapy.

#### 3.3.3. Cell Viability

**Figure 9:**
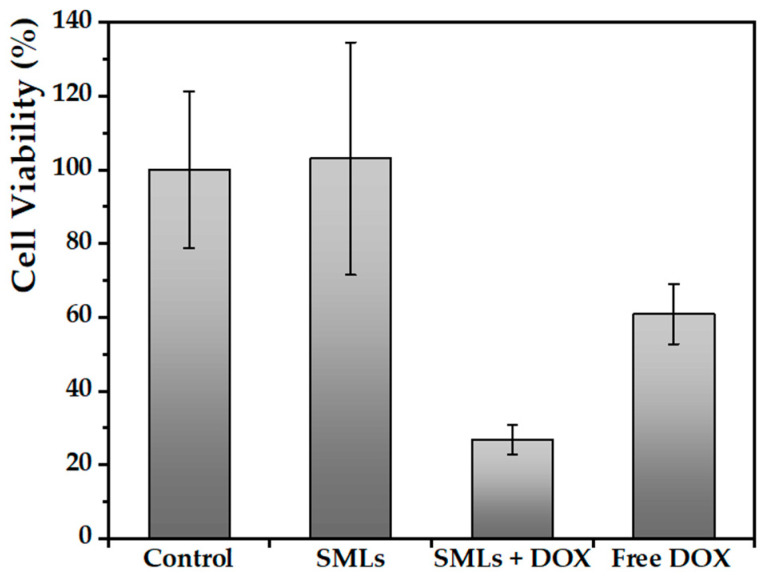
Viability of untreated HepG2 cancer cells (control) and in the presence of solid magnetoliposomes without DOX (SMLs) and with DOX encapsulated at 5.65 × 10^−5^ M (SMLs + DOX), as well as in the presence of drug in free form at 5.65 × 10^−5^ M (Free DOX).

Preliminary biological studies were carried out by evaluating the effect of DOX-loaded solid magnetoliposomes on the cancer cell line HepG2. For this, viability assays were performed incubating the cells with drug-loaded SMLs at 5.65 × 10^−5^ M. For comparison purposes, free doxorubicin was also tested (Figure 9). After the 48-hour assay, the viability of cells subjected to SMLs without DOX increased due to higher cellular proliferation than in the control, which is indicative of the development of a safe and non-toxic nanosystem. In all other situations, there was a great decrease in cell viability. In this study, it is highlighted that the developed formulation of pH-sensitive SMLs seems to promote drug entry into the cells, this nanosystem being more effective in cancer therapy compared to the chemotherapeutic agent alone. A maximum inhibitory activity of approximately 73.2% was observed at this low concentration of DOX. In order to determine the effect of the antineoplastic agent concentration on cell viability, a higher DOX concentration was tested (Figure S3—Supplementary Material). However, the differences in inhibitory activity obtained are not very significant, reinforcing the fact that there is no need for using a high drug concentration to obtain a notable effect in tumor cells. Based on the previously presented DOX release profile, it is proven that these SMLs effectively interact with cells, allowing a more efficient release of the drug in acidic media. All of the above has a great influence on the toxicity exhibited by these nanosystems.

Thus, it is possible to predict an even more pronounced reduction in cell viability by subjecting tumor cells to DOX-loaded SMLs under the application of an alternating magnetic field and/or a laser light source, exercising a dual therapeutic approach. In these assays, HepG2 cells did not show any cellular activity, which strongly demonstrates the importance of developing magnetic nanosystems sensitive to specific stimuli of the tumor microenvironment.

## 4. Conclusions

In this work, shape anisotropic Mg_0.75_Ca_0.25_Fe_2_O_4_ magnetic nanoparticles were prepared and characterized in detail. The magnetic properties allowed characterizing the nanoparticles as superparamagnetic, an ideal behavior for application as magnetic hyperthermia agents. Magnetic hyperthermia (SAR = 4.98 W/g) and photothermal (SAR = 1.1 × 10^4^ W/g) measurements confirmed the enhanced potential of nanorods in cancer treatment. The calcined nanoparticles, with rod-like shape, were selected as the magnetic component of pH-sensitive DOPE:Ch:CHEMS (45:45:10) SMLs. After the effective incorporation of the drug doxorubicin in the SMLs, with a high encapsulation efficiency, its release profile was studied, which was much more pronounced in acidic medium (55%). Biological assays on the HepG2 cell line confirmed the drug delivery capability of SMLs, reducing cancer cells’ viability to 26.8% using a low doxorubicin concentration, and the development of a safe and non-toxic nanosystem was confirmed.

Considering all the results, the combination of non-spherical magnetic nanoparticles with a pH-sensitive liposomal formulation emerges as a promising approach to exert a multimodal cancer therapy through pH-sensitive drug delivery combined with dual hyperthermia (magnetic and photothermia). To the best of our knowledge, the pH-sensitive DOPE:Ch:CHEMS (45:45:10) formulation is innovative in the composition of solid magnetoliposomes.

## Figures and Tables

**Table 1:** Parameters obtained by Rietveld analysis of calcined Mg_0.75_Ca_0.25_Fe_2_O_4_ magnetic nanoparticles. Calculated R_P_ and χ^2^ parameters, phase sizes, strain and degree of inversion obtained by analysis of X-ray diffraction pattern using Profex software.

O_x,y,z_ (a)	*i* (b)	Phase Size (nm)	Lattice Constant (nm)	R_P_	χ^2^
0.3769 ± 0.0009	0.825	18.7 ± 0.3	0.8367 ± 0.0001	8.67	1.46
0.3761 ± 0.0009	0.675 ± 0.02			8.62	1.44

(a) Value of O_x,y,z_ in CIF file is 0.375. (b) *i*—degree of inversion.

**Table 2:** Results of SQUID analysis at 300 K: Coercivity (H_C_), saturation magnetization (M_s_), remnant magnetization (M_r_) and squareness value (M_r_/M_s_) for non-calcined and calcined Mg_0.75_Ca_0.25_Fe_2_O_4_ nanoparticles.

MNPs	H_C_ (Oe)	M_s_ (emu/g)	M_r_ (emu/g)	M_r_/M_s_
Non-calcined	5.72	35.92	0.52	0.014
Calcined	19.49	45.21	1.74	0.038

**Table 3:** Slope (ΔT/Δt), coefficient of determination (R^2^), nanoparticles’ mass (m_MNPs_) and SAR value obtained by magnetic and photothermal hyperthermia.

Hyperthermia	ΔT/Δt	R^2^	m_MNPs_ (mg)	SAR (W/g)
Magnetic	1.2143	0.8436	24.97	203.27
Photothermal	2.4786	0.9593	1.04	11021.86

**Table 4:** Hydrodynamic diameter (nm), polydispersity index (PDI) and zeta potential (mV) and respective standard deviation (SD) in acidic (pH = 5) and physiological (pH = 7.4) conditions of the liposomal formulations DOPE:CHEMS (7:3), DOPE:Ch:CHEMS (45:45:10) and DPPE:Ch:CHEMS (45:45:10).

Lipid Formulation	pH	Hydrodynamic Diameter ± SD (nm)	PDI ± SD	Zeta Potential ± SD (mV)
DOPE:CHEMS	5	*	0.31 ± 0.007	−8.39 ± 1.05
	7.4	101.43 ± 0.63	0.24 ± 0.004	−17.19 ± 0.95
DOPE:Ch:CHEMS	5	*	0.30 ± 0.03	−7.93 ± 0.51
	7.4	161.90 ± 5.22	0.26 ± 0.004	−22.78 ± 0.92
DPPE:Ch:CHEMS	5	*	0.40 ± 0.003	−11.69 ± 1.42
	7.4	360.01 ± 132.75	0.39 ± 0.07	−25.10 ± 1.22

* Outside the device’s detection limit.

**Table 5:** Hydrodynamic diameter (nm), polydispersity index (PDI) and zeta potential (mV) and respective SD of liposomes of DOPE:Ch:CHEMS (45:45:10) and SMLs containing the same lipids and calcined Mg_0.75_Ca_0.25_Fe_2_O_4_ nanoparticles, at pH = 7.4.

Nanosystem	Hydrodynamic Diameter ± SD (nm)	PDI ± SD	Zeta Potential ± SD (mV)
Liposomes	161.9 ± 5.2	0.26 ± 0.0004	−22.78 ± 0.92
SMLs	149.4 ± 24.2	0.15 ± 0.01	−28.19 ± 1.68

**Table 6:** Doxorubicin encapsulation efficiency, EE(%), in DOPE:Ch:CHEMS (45:45:10) SMLs containing Mg_0.75_Ca_0.25_Fe_2_O_4_ nanoparticles from three independent measurements (i, ii and iii) and respective mean and standard deviation.

Assay	EE(%)	Mean ± SD (%)
i	99.22	98.71 ± 0.97
ii	97.32	
iii	99.59	

**Table 7:** Parameters obtained by fitting the mathematical models to the release kinetics of doxorubicin encapsulated in SMLs (Weibull and first-order) and respective coefficients of determination (R^2^) for each pH value studied (5 and 7.4).

pH	Weibull			First-Order	
	B	A	R^2^	k (min^−1^)	R^2^
5	0.432	8.73 × 10^−5^	0.993	0.121	0.974
7.4	0.0324	0.0111	0.992	0.0542	0.980

## Data Availability

Not applicable.

## Supplementary Materials

The following supporting information can be downloaded at: https://www.mdpi.com/article/10.3390/nano13061051/s1, Table S1: SAR values obtained by photothermal hyperthermia of different nanoparticles under a NIR laser at λ = 808 nm and power density of 1 W/cm^2^; Figure S1: TEM image of solid magnetoliposomes containing a DOPE:Ch:CHEMS (45:45:10) lipid bilayer; Figure S2: Variation, for a storage period of 7 days, of: (a) hydrodynamic diameter (blue) and PDI (red); and (b) zeta potential (green) of an aqueous solution of DOPE:Ch:CHEMS (45:45:10) SMLs at pH=7.4; Figure S3: Viability of HepG2 cancer cells in the presence of DOX-loaded solid magnetoliposomes at 1.13 × 10^−4^ M (SMLs [+]), as well as in the presence of drug in free form at the same concentration (DOX [+]).

## References

[1] Chaturvedi V.K., Singh A., Singh V.K., Singh M.P. (2019). Cancer Nanotechnology: A New Revolution for Cancer Diagnosis and Therapy. Curr. Drug Metab..

[2] Ahmed S., Rajak B.L., Gogoi M., Sarma H.D., Paul S., Bhatia D. (2020). Magnetic nanoparticles mediated cancer hyperthermia. Smart Healthcare for Disease Diagnosis and Prevention.

[3] Rodrigues A.R.O., Mendes P.M.F., Silva P.M.L., Machado V.A., Almeida B.G., Araújo J.P., Queiroz M.J.R.P., Castanheira E.M.S., Coutinho P.J.G. (2017). Solid and aqueous magnetoliposomes as nanocarriers for a new potential drug active against breast cancer. Colloids Surf. B.

[4] Ansari M., Eslami H., Javadpour S., Yousefi E. (2021). Cancer Therapy Using a Targeted Magnetoliposomes Encapsulated Doxorubicin Assisted Ultrasound. Mater. Technol..

[5] Guo Y., Zhang Y., Ma J., Li Q., Li Y., Zhou X., Zhao D., Song H., Chen Q., Zhu X. (2018). Light/magnetic hyperthermia triggered drug released from multi-functional thermo-sensitive magnetoliposomes for precise cancer synergetic theranostics. J. Control. Release.

[6] Wang E.C., Wang A.Z. (2014). Nanoparticles and their applications in cell and molecular biology. Integr. Biol..

[7] Espinosa A., Di Corato R., Kolosnjaj-Tabi J., Flaud P., Pellegrino T., Wilhelm C. (2016). Duality of Iron Oxide Nanoparticles in Cancer Therapy: Amplification of Heating Efficiency by Magnetic Hyperthermia and Photothermal Bimodal Treatment. ACS Nano.

[8] Vinícius-Araújo M., Shrivastava N., Sousa-Junior A.A., Mendanha S.A., Santana R.C.D., Bakuzis A.F. (2021). Zn_x_Mn_1-x_Fe_2_O_4_@SiO_2_: Z Nd^3+^ Core–Shell Nanoparticles for Low-Field Magnetic Hyperthermia and Enhanced Photothermal Therapy with the Potential for Nanothermometry. ACS Appl. Nano Mater..

[9] Gandhi S., Issar S., Mahapatro A.K., Roy I. (2020). Cobalt ferrite nanoparticles for bimodal hyperthermia and their mechanistic interactions with lysozyme. J. Mol. Liq..

[10] Alotaibi I., Alshammari M.S., Algessair S., Madkhali N., All N.A., Hjiri M., Alrub S.A., Mir L.E., Lemine O.M. (2022). Synthesis, characterization and heating efficiency of Gd-doped maghemite (γ-Fe_2_O_3_) nanoparticles for hyperthermia application. Physica B Condens. Matter.

[11] Plichta Z., Kozak Y., Panchuk R., Sokolova V., Epple M., Kobylinska L., Jendelová P., Horák D. (2018). Cytotoxicity of doxorubicin-conjugated poly[*N*-(2-hydroxypropyl)methacrylamide]-modified γ-Fe_2_O_3_ nanoparticles towards human tumor cells. Beilstein J. Nanotech..

[12] Dutta B., Shetake N.G., Gawali S.L., Barick B.K., Barick K.C., Babu P.D., Pandey B.N., Priyadarsini K.I., Hassan P.A. (2018). PEG mediated shape-selective synthesis of cubic Fe_3_O_4_ nanoparticles for cancer therapeutics. J. Alloys Compd..

[13] Sirivat A., Paradee N. (2019). Facile synthesis of gelatin-coated Fe_3_O_4_ nanoparticle: Effect of pH in single-step co-precipitation for cancer drug loading. Mater. Des..

[14] Fu S., Wang S., Zhang X., Qi A., Liu Z., Yu X., Chen C., Li L. (2017). Structural effect of Fe_3_O_4_ nanoparticles on peroxidase-like activity for cancer therapy. Colloids Surf. B.

[15] Pereira D.S.M., Cardoso B.D., Rodrigues A.R.O., Amorim C.O., Amaral V.S., Almeida B.G., Queiroz M.J.R.P., Martinho O., Baltazar F., Calhelha R.C. (2019). Magnetoliposomes Containing Calcium Ferrite Nanoparticles for Applications in Breast Cancer Therapy. Pharmaceutics.

[16] Purushothaman B.K., Maheswari P.U., Begum K.M.M.S. (2021). pH and magnetic field responsive protein-inorganic nanohybrid conjugated with biotin: A biocompatible carrier system targeting lung cancer cells. J. Appl. Polym. Sci..

[17] Bilas R., Sriram K., Maheswari P.U., Begum K.M.M.S. (2017). Highly biocompatible chitosan with super paramagnetic calcium ferrite (CaFe_2_O_4_) nanoparticle for the release of ampicillin. Int. J. Biol. Macromol..

[18] Nigam A., Saini S., Singh B., Rai A.K., Pawar S.J. (2022). Zinc doped magnesium ferrite nanoparticles for evaluation of biological properties viz antimicrobial, biocompatibility, and in vitro cytotoxicity. Mater. Today Commun..

[19] Meidanchi A., Motamed A. (2020). Preparation, characterization and in vitro evaluation of magnesium ferrite superparamagnetic nanoparticles as a novel radiosensitizer of breast cancer cells. Ceram. Int..

[20] Maehara T., Konishi K., Kamimori T., Aono H., Hirazawa H., Naohara T., Nomura S., Kikkawa H., Watanabe Y., Kawachi K. (2005). Selection of ferrite powder for thermal coagulation therapy with alternating magnetic field. J. Mater. Sci..

[21] Hirazawa H., Kusamoto S., Aono H., Naohara T., Mori K., Hattori Y., Maehara T., Watanabe Y. (2008). Preparation of fine Mg_1−x_Ca_x_Fe_2_O_4_ powder using reverse coprecipitation method for thermal coagulation therapy in an ac magnetic field. J. Alloys Compd..

[22] Yougbaré S., Chou H.L., Yang C.H., Krisnawati D.I., Jazidie A., Nuh M., Kuo T.R. (2021). Facet-dependent gold nanocrystals for effective photothermal killing of bacteria. J. Hazard. Mater..

[23] Cardoso B.D., Rodrigues A.R.O., Bañobre-López M., Almeida B.G., Amorim C.O., Amaral V.S., Coutinho P.J.G., Castanheira E.M.S. (2021). Magnetoliposomes Based on Shape Anisotropic Calcium/Magnesium Ferrite Nanoparticles as Nanocarriers for Doxorubicin. Pharmaceutics.

[24] Arjama M., Mehnath S., Jeyaraj M. (2022). Self-assembled hydrogel nanocube for stimuli responsive drug delivery and tumor ablation by phototherapy against breast cancer. Int. J. Biol. Macromol..

[25] Panda S., Bhol C.S., Bhutia S.K., Mohapatra S. (2021). PEG–PEI-modified gated N-doped mesoporous carbon nanospheres for pH/NIR light-triggered drug release and cancer phototherapy. J. Mater. Chem. B.

[26] Lisjak D., Mertelj A. (2018). Anisotropic magnetic nanoparticles: A review of their properties, syntheses and potential applications. Prog. Mater. Sci..

[27] Andrade R.G.D., Veloso S.R.S., Castanheira E.M.S. (2020). Shape Anisotropic Iron Oxide-Based Magnetic Nanoparticles: Synthesis and Biomedical Applications. Int. J. Mol. Sci..

[28] Roca A.G., Gutiérrez L., Gavilán H., Brollo M.E.F., Veintemillas-Verdaguer S., Morales M.P. (2019). Design strategies for shape-controlled magnetic iron oxide nanoparticles. Adv. Drug Deliv. Rev..

[29] Lombardo D., Calandra P., Pasqua L., Magazù S. (2020). Self-Assembly of Organic Nanomaterials and Biomaterials: The Bottom-Up Approach for Functional Nanostructures Formation and Advanced Applications. Materials.

[30] Aghdam M.A., Bagheri R., Mosafer J., Baradaran B., Hashemzaei M., Baghbanzadeh A., de la Guardia M., Mokhtarzadeh A. (2019). Recent advances on thermosensitive and pH-sensitive liposomes employed in controlled release. J. Control. Release.

[31] El-Sawy H.S., Al-Abd A.M., Ahmed T.A., El-Say K.M., Torchilin V.P. (2018). Stimuli-Responsive Nano-Architecture Drug-Delivery Systems to Solid Tumor Micromilieu: Past, Present, and Future Perspectives. ACS Nano.

[32] Ferreira D.S., Lopes S.C.A., Franco M.S., Oliveira M.C. (2013). pH-sensitive liposomes for drug delivery in cancer treatment. Ther. Deliv..

[33] Silva J.O., Fernandes R.S., Oda C.M.R., Ferreira T.H., Botelho A.F.M., Melo M.M., de Miranda M.C., Gomes D.A., Cassali G.D., Townsend D.M. (2019). Folate-coated, long-circulating and pH-sensitive liposomes enhance doxorubicin antitumor effect in a breast cancer animal model. Biomed. Pharmacother..

[34] Yao Y., Wang T., Liu Y., Zhang N. (2019). Co-delivery of sorafenib and VEGF-siRNA via pH-sensitive liposomes for the synergistic treatment of hepatocellular carcinoma. Artif. Cells Nanomed. Biotechnol..

[35] Rehman A.U., Omran Z., Anton H., Mély Y., Akram S., Vandamme T.F., Anton N. (2018). Development of doxorubicin hydrochloride loaded pH-sensitive liposomes: Investigation on the impact of chemical nature of lipids and liposome composition on pH-sensitivity. Eur. J. Pharm. Biopharm..

[36] Gouveia V.A.M. (2014). pH sensitive liposomes for the treatment of rheumatoid arthritis. Master’s Thesis.

[37] Hafez I.M., Cullis P.R. (2000). Cholesteryl hemisuccinate exhibits pH sensitive polymorphic phase behavior. Biochim. Et Biophys. Acta (BBA)-Biomembr..

[38] Li S., Qin G.W., Pei W., Ren Y., Zhang Y., Esling C., Zuo L. (2009). Capping Groups Induced Size and Shape Evolution of Magnetite Particles Under Hydrothermal Condition and their Magnetic Properties. J. American Cer. Society.

[39] Humphrey J.J.L., Sadasivan S., Plana D., Celorrio V., Tooze R.A., Fermín D.J. (2015). Surface Activation of Pt Nanoparticles Synthesised by “Hot Injection” in the Presence of Oleylamine. Chem.—Eur. J..

[40] Gouda A., Sakr O.S., Nasr M., Sammour O. (2021). Ethanol injection technique for liposomes formulation: An insight into development, influencing factors, challenges and applications. J. Drug Deliv. Sci. Technol..

[41] Li J., Wang X., Zhang T., Wang C., Huang Z., Luo X., Deng Y. (2015). A review on phospholipids and their main applications in drug delivery systems. Asian J. Pharm. Sci..

[42] Iacovita C., Florea A., Dudric R., Pall E., Moldovan A.I., Tetean R., Stiufiuc R., Lucaciu C.M. (2016). Small versus Large Iron Oxide Magnetic Nanoparticles: Hyperthermia and Cell Uptake Properties. Molecules.

[43] Phong P.T., Nam P.H., Manh D.H., Lee I.J. (2017). Mn_0.5_Zn_0.5_Fe_2_O_4_ nanoparticles with high intrinsic loss power for hyperthermia therapy. J. Magn. Magn. Mater..

[44] Zhang X., Niu Y., Li Y., Hou X., Wang Y., Bai R., Zhao J. (2013). Synthesis, optical and magnetic properties of α-Fe_2_O_3_ nanoparticles with various shapes. Mater. Lett..

[45] Cardoso B.D., Rodrigues A.R.O., Almeida B.G., Amorim C.O., Amaral V.S., Castanheira E.M.S., Coutinho P.J.G. (2020). Stealth Magnetoliposomes Based on Calcium-Substituted Magnesium Ferrite Nanoparticles for Curcumin Transport and Release. Int. J. Mol. Sci..

[46] Wu K., Su D., Liu J., Saha R., Wang J.P. (2019). Magnetic nanoparticles in nanomedicine: A review of recent advances. Nanotechnology.

[47] Döbelin N., Kleeberg R. (2015). Profex: A graphical user interface for the Rietveld refinement program BGMN. J. Appl. Crystallog..

[48] Bergmann J., Friedel P., Kleeberg R. (1998). IUCr Commission on Powder Diffraction. Newsletter.

[49] Cardoso B.D., Rio I.S.R., Rodrigues A.R.O., Fernandes F.C.T., Almeida B.G., Pires A., Pereira A.M., Araujo J.P., Castanheira E.M.S., Coutinho P.J.G. (2018). Magnetoliposomes containing magnesium ferrite nanoparticles as nanocarriers for the model drug curcumin. Royal Soc. Open Sci..

[50] Kumar L., Kumar P., Narayan A., Kar M. (2013). Rietveld analysis of XRD patterns of different sizes of nanocrystalline cobalt ferrite. Int. Nano Lett..

[51] Cui K.K., Wu Z.J., Huang W., Gao Z.F., Shen X.M., Liu W.M. (2014). Recycle of Valuable Metals in Converter Steel Slag for Preparing Multidoped M_x_Mg_1-x_ Fe_2_O_4_ (M = Mn, Ca) Spinel. ACS Sustain. Chem. Eng..

[52] Riaz M.K., Riaz M.A., Zhang X., Lin C., Wong K.H., Chen X., Zhang G., Lu A., Yang Z. (2018). Surface Functionalization and Targeting Strategies of Liposomes in Solid Tumor Therapy: A Review. Int. J. Mol. Sci..

[53] Immordino M.L., Dosio F., Cattel L. (2006). Stealth liposomes: Review of the basic science, rationale, and clinical applications, existing and potential. Int. J. Nanomed..

[54] Rodrigues A.R.O., Ramos J.M., Gomes I.T., Almeida B.G., Araújo J.P., Queiroz M.J.R.P., Coutinho P.J.G., Castanheira E.M.S. (2016). Magnetoliposomes based on manganese ferrite nanoparticles as nanocarriers for antitumor drugs. RSC Adv..

[55] Yoo D., Lee J.H., Shin T.H., Cheon J. (2011). Theranostic Magnetic Nanoparticles. Acc. Chem. Res..

[56] Song Q., Zhang J. (2004). Shape Control and Associated Magnetic Properties of Spinel Cobalt Ferrite Nanocrystals. J. Am. Chem. Soc..

[57] Das R., Alonso J., Porshokouh Z.N., Kalappattil V., Torres D., Phan M.H., Garaio E., García J.A., Llamazares J.L.S., Srikanth H. (2016). Tunable High Aspect Ratio Iron Oxide Nanorods for Enhanced Hyperthermia. J. Phys. Chem. C.

[58] Liu Y., Guo Z., Li F., Xiao Y., Zhang Y., Bu T., Jia P., Zhe T., Wang L. (2019). Multifunctional Magnetic Copper Ferrite Nanoparticles as Fenton-like Reaction and Near-Infrared Photothermal Agents for Synergetic Antibacterial Therapy. ACS Appl. Mater. Interfaces.

[59] Gupta R., Sharma D. (2020). Manganese-Doped Magnetic Nanoclusters for Hyperthermia and Photothermal Glioblastoma Therapy. ACS Appl. Nano Mater..

[60] Espinosa A., Kolosnjaj-Tabi J., Abou-Hassan A., Sangnier A.P., Curcio A., Silva A.K.A., Corato R.D., Neveu S., Pellegrino T., Liz-Marzán L.M. (2018). Magnetic (Hyper)Thermia or Photothermia? Progressive Comparison of Iron Oxide and Gold Nanoparticles Heating in Water, in Cells, and In Vivo. Adv. Funct. Mater..

[61] Danaei M., Dehghankhold M., Ataei S., Davarani F.H., Javanmard R., Dokhani A., Khorasani S., Mozafari M.R. (2018). Impact of Particle Size and Polydispersity Index on the Clinical Applications of Lipidic Nanocarrier Systems. Pharmaceutics.

[62] Ribeiro B.C., Alvarez C.A.R., Alves B.C., Rodrigues J.M., Queiroz M.J.R.P., Almeida B.G., Pires A.L., Pereira A., Araújo J.P., Coutinho P.J.G. (2022). Development of Thermo- and pH-Sensitive Liposomal Magnetic Carriers for New Potential Antitumor Thienopyridine Derivatives. Materials.

[63] Dash S., Murthy P.N., Nath L., Chowdhury P. (2010). Kinetic modeling on drug release from controlled drug delivery systems. Acta Pol. Pharm..

[64] Moussout H., Ahlafi H., Aazza M., Maghat H. (2018). Critical of linear and nonlinear equations of pseudo-first order and pseudo-second order kinetic models. Karbala Int. J. Mod. Sci..

[65] Papadopoulou V., Kosmidis K., Vlachou M., Macheras P. (2006). On the use of the Weibull function for the discernment of drug release mechanisms. Int. J. Pharm..

