# Supplementary material for: Development of pH-Sensitive Magnetoliposomes Containing Shape Anisotropic Nanoparticles for Potential Application in Combined Cancer Therapy
Source: Nanomaterials (Basel). 2023 Mar 15;13(6):1051. doi: 10.3390/nano13061051 (PMC10054438; doi:10.3390/nano13061051)
Supplement: Supplementary file 1 [file nanomaterials-13-01051-s001.zip › nanomaterials-2258719-supplementary.pdf]

Development of pH-sensitive magnetoliposomes containing shape anisotropic nanoparticles for potential application in combined cancer therapy

Supplementary Material

1. Photothermal hyperthermia efficiency

Table S1. SAR values obtained by photothermal hyperthermia of different nanoparticles under a NIR laser at λ = 808 nm and power density of 1 W/cm^2 [60].

MNPs Composition	SAR (W/g)
γ-Fe_2O_3 rock-like NPs	~ 200
$CoFe_2O_4$ NPs	~ 650
γ-Fe_3O_4 nanocubes	1100

2. SMLs TEM image and colloidal stability

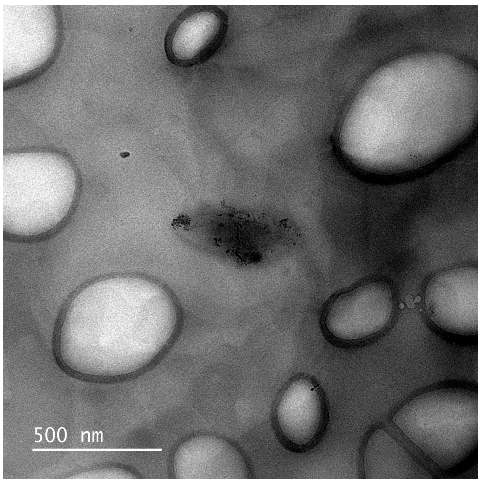

Figure S1. TEM image of solid magnetoliposomes containing a DOPE:Ch:CHEMS (45:45:10) lipid bilayer.

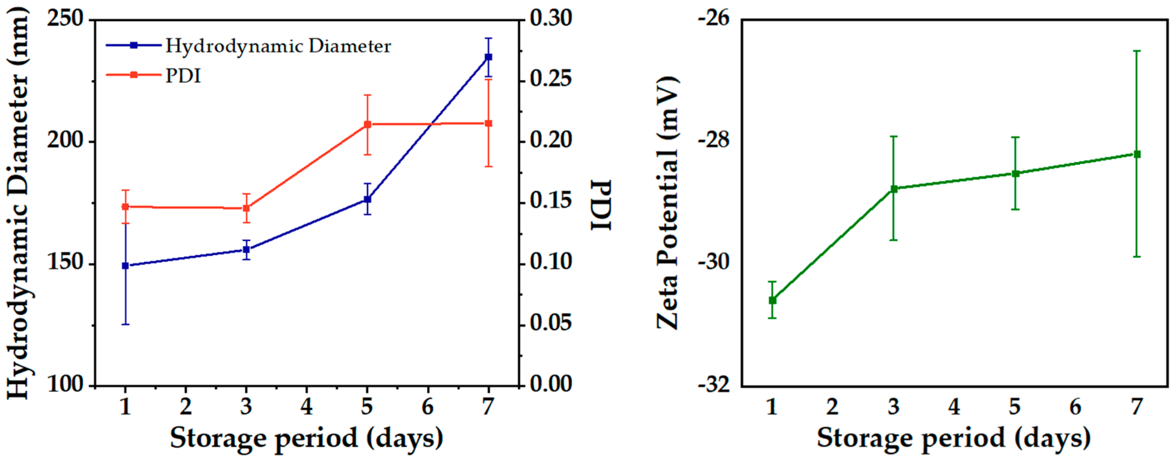

(**a**) (**b**)

Figure S2. Variation, for a storage period of 7 days, of: (**a**) hydrodynamic diameter (blue) and PDI (red); and (**b**) zeta potential (green) of an aqueous solution of DOPE:Ch:CHEMS (45:45:10) SMLs at pH=7.4.

3. Cell viability

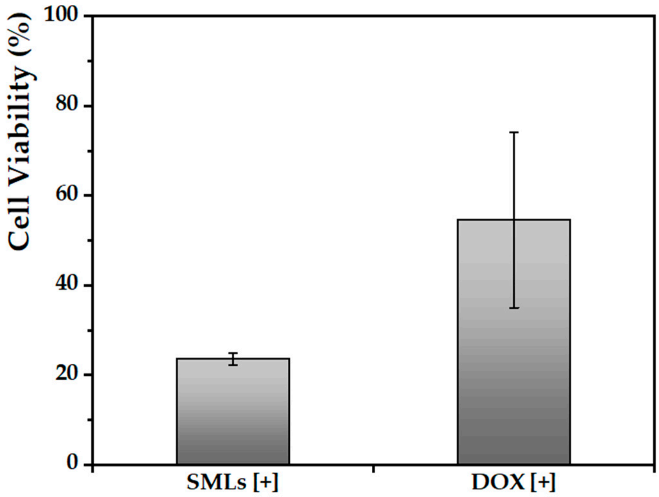

Figure S3. Viability of HepG2 cancer cells in the presence of DOX-loaded solid magnetoliposomes at 1.13 x 10^{-4} M (SMLs [+]), as well as in the presence of drug in free form at the same concentration (DOX [+]).

References

[60] Espinosa, A.; Kolosnjaj-Tabi, J.; Abou-Hassan, A.; Sangnier, A.P.; Curcio, A.; Silva, A.K.A.; Corato, R.D.; Neveu, S.; Pellegrino, T.; Liz-Marzán, L.M.; Wilhelm, C. Magnetic (Hyper)Thermia or Photothermia? Progressive Comparison of Iron Oxide and Gold Nanoparticles Heating in Water, in Cells, and In Vivo. *Adv. Func. Mater.* **2018**, *28*, 1803660. [DOI: 10.1002/adfm.201803660]
